# A multicentre comparison of quantitative ^90^Y PET/CT for dosimetric purposes after radioembolization with resin microspheres: The QUEST Phantom Study

**DOI:** 10.1007/s00259-015-3059-9

**Published:** 2015-05-13

**Authors:** Kathy P. Willowson, Michael Tapner, The QUEST Investigator Team, Dale L. Bailey

**Affiliations:** Institute of Medical Physics, School of Physics, University of Sydney, Sydney, 2006 NSW Australia; Sirtex, North Sydney, 2060 NSW Australia; Department of Nuclear Medicine, Royal North Shore Hospital, St Leonards, Sydney, 2065 NSW Australia; Faculty of Health Sciences, University of Sydney, Lidcombe, 2141 Australia

**Keywords:** Yttrium-90, PET/CT, Quantitative, Radioembolization

## Abstract

**Purpose:**

To investigate and compare the quantitative accuracy of ^90^Y imaging across different generation PET/CT scanners, for the purpose of dosimetry after radioembolization with resin microspheres.

**Methods:**

A strict experimental and imaging protocol was followed by 47 international sites using the NEMA 2007/IEC 2008 PET body phantom with an 8-to-1 sphere-to-background ratio of ^90^Y solution. The phantom was imaged over a 7-day period (activity ranging from 0.5 to 3.0 GBq) and all reconstructed data were analysed at a core laboratory for consistent processing. Quantitative accuracy was assessed through measures of total phantom activity, activity concentration in background and hot spheres, misplaced counts in a nonradioactive insert, and background variability.

**Results:**

Of the 69 scanners assessed, 37 had both time-of-flight (ToF) and resolution recovery (RR) capability. These current generation scanners from GE, Philips and Siemens could reconstruct background concentration measures to within 10 % of true values over the evaluated range, with greater deviations on the Philips systems at low count rates, and demonstrated typical partial volume effects on hot sphere recovery, which dominated spheres of diameter <20 mm. For spheres >20 mm in diameter, activity concentrations were consistently underestimated by about 20 %. Non-ToF scanners from GE Healthcare and Siemens were capable of producing accurate measures, but with inferior quantitative recovery compared with ToF systems.

**Conclusion:**

Current generation ToF scanners can consistently reconstruct ^90^Y activity concentrations, but they underestimate activity concentrations in small structures (≤37 mm diameter) within a warm background due to partial volume effects and constraints of the reconstruction algorithm. At the highest count rates investigated, measures of background concentration (about 300 kBq/ml) could be estimated on average to within 1 %, 5 % and 2 % for GE Healthcare (all-pass filter, RR + ToF), Philips (4i8s ToF) and Siemens (2i21s all-pass filter, RR + ToF) ToF systems, respectively. Over the range of activities investigated, comparable performance between GE Healthcare and Siemens ToF systems suggests suitability for quantitative analysis in a scenario analogous to that of postradioembolization imaging for treatment of liver cancer.

## Introduction

Combined PET and CT (PET/CT) imaging of ^90^Y microspheres is fast becoming part of the routine protocol to confirm accurate delivery of radionuclide therapy to tumours in the liver after radioembolization. Clinical affirmation of the PET/CT imaging technique was first published in 2009 [1], and relies on the minute positron branching ratio (with probability 31.86 ± 0.47 × 10^−6^ [2]) as a result of pair production following de-excitation from the 0^+^excited state of ^90^Zr [3]. Since that time its clinical use has grown steadily, ranging from confirmation of radionuclide targeting and absence of extrahepatic uptake [4, 5], to activity quantification for dosimetry [6–11].

Whilst ^90^Y PET/CT is a desirable tool for assessment of the efficacy of the radioembolization procedure, there is much that is not well understood about the effects of the physical decay characteristics on the imaging and reconstruction process. This includes the impact of the low true coincidence counting rate due to the low positron branching ratio which results in noisy image data. In addition, such a low true coincidence rate means that the prompt gamma emissions from the natural ^176^Lu in LSO/LYSO crystals of certain PET scanners cannot necessarily be ignored, as is the case with typical PET radionuclides which have true coincidence rates that are orders of magnitude greater. Furthermore, the large flux of bremsstrahlung photons from the dominant beta decay mode of ^90^Y results in a singles count rate that exceeds the true coincidence count rate by a large factor, which was originally thought to have potential for detector saturation when high amounts of ^90^Y activity are imaged [1], although this has not been found in more recent investigations (for example [7, 4, 12]). The additional bremsstrahlung photons and prompt gammas result in a very high random fraction when imaging ^90^Y, seen to be in the order of 80 % at our institution, compared to a typical FDG scan of 30 – 40 %. Combined with problematic scatter modelling for such low count data, this typically results in very noisy true coincidence sinograms following the subtraction of both scatter and random events, which will ultimately affect both the qualitative and quantitative aspect of the reconstruction.

Postradioembolization ^90^Y PET/CT has the potential to allow an improved understanding of the absorbed dose–response relationship on a cancer-specific basis, information which may be used in the future to tailor treatments specifically to the individual. In order to establish a meaningful association between absorbed dose and response, a large-scale study is necessary, recruiting significant numbers of patients who are typically not available from any one site. Such a multicentre trial relies heavily on the comparability of intersite data, which relies on the quantitative accuracy and comparability of the imaging equipment itself [13]. The idea of *harmonization* of the image acquisition and analysis approach to establish intersite compatibility for multicentre trials based on initial phantom studies has been explored in the literature (for example [14, 15]). Makris et al. [16] found that the standard NEMA NU-2 image quality phantom is ideal for intersite testing and looking for differences in quantitative concentration measures, and that comparison of the quantitative accuracy of ^18^F imaging is better achieved using an average concentration measure across a volume, as opposed to a maximum. Geworski et al. [17] found that errors in FDG standardized uptake value (SUV) measurement (performed by a single observer) across multisite PET scanners using a uniformly filled phantom were below 10 % in 15 out of 19 tested scanners (3D imaging), in agreement with the findings of Park et al. [18] who also derived SUV calibration values for each system which could be applied to intersite compatibility of measures, with a maximum reported variation corresponding to a calibration factor of 1.24 (i.e. a 24 % variation in measurement). Whilst a number of phantom studies have been performed with ^90^Y on current generation scanners [12, 19–23], to date there are no data to suggest that quantification estimates from all PET scanners are optimized and accurate (particularly when compared with known scanner performance with FDG), or that they are consistent across different generations and vendors, so as to offer comparable data in a trial setting.

The objective of this study was to investigate and compare the quantitative accuracy of ^90^Y PET/CT imaging on a large number of scanners from multiple sites, with the specific intention of moving towards a uniform approach in the setting of a large-scale clinical trial to establish the absorbed dose–response relationship following radioembolization with ^90^Y SIR-Spheres microspheres (Sirtex, Sydney, Australia) for liver cancer. As such, this report represents the preclinical assessment phase of a larger collaboration led by The University of Sydney, The Royal North Shore Hospital, and Sirtex (known as QUEST—Quantitative Uptake Evaluation in SIR-Spheres Therapy).

## Materials and methods

Data were acquired on a variety of PET scanners (Table 1) from the major vendors, with a number of different reconstructions from systems equipped both with and without time of flight (ToF) and resolution recovery (RR). Each site followed an identical experimental protocol utilizing the NEMA 2007/IEC 2008 PET Body Phantom (Data Spectrum Corporation, NC), with a volume of about 10 L containing a “cold” (nonradioactive) solid insert (diameter 51 mm) and six fillable spheres of various diameters (∅ 10, 13, 17, 22, 28 and 37 mm) filled to an approximate eight-to-one sphere-to-background ratio with ^90^Y-chloride (YCl_3_) provided in a constant specific activity (PerkinElmer, Waltham, MA).

**Table 1:** The scanners contributing data to the study according to vendor and model (all scanners equipped with standard reconstruction corrections for attenuation, scatter and random events)

Vendor	Model	Crystal material	Additional corrections	Number of scanners	Number of reconstructions
GE Healthcare	Discovery 690, 710	LYSO	ToF, RR	9	21
	Discovery 600, Discovery ST (E)	BGO	With or without RR	9	16
	Discovery RX	LYSO	–	3	7
Philips	Gemini TF	LYSO	ToF, RR	9	9
Siemens	Biograph mCT	LSO	ToF, RR	19^a^	28
	Biograph (various)	LSO	With or without RR	19	28

*ToF’* time-of-flight, *RR’* resolution recovery (point spread function recovery)

^a^Including two systems with the new continuous bed motion technology

Each site was required to measure the phantom volume and the delivered ^90^Y solution in the departmental dose calibrator for comparison with the shipping certificate. The entire delivery vial was added to a volume of 1,300 ml, and this solution was used to fill the phantom spheres, before the reminder of solution was added to the background compartment, with the addition of EDTA/DTPA to the contents to prevent the YCl_3_ sticking to the phantom walls. This allowed an eight-to-one sphere-to-background ratio, in keeping with the NEMA NU 2-2007 [24] image quality guidelines, and was thought to require minimal phantom manipulation and activity handling at sites. Residual in the vial was estimated through re-measuring the vial in the dose calibrator after reconstitution to the initial volume with water. Residual in the needle and syringe was taken as negligible.

### Imaging and reconstruction

After filling with [^90^Y]YCl_3_ according to the instructions supplied, the phantom was imaged on days 0, 3, 5 and 7, during which time the total activity decayed from 3 GBq to 0.5 GBq, thus covering the recommended activity range for therapy prescribed in the SIR-Spheres package insert formula. This was done to assess scanner performance under different rates of photon fluence, and to assess the impact of background radiation from ^176^Lu present in current generation (LSO/LYSO) detector crystals at lower counting rates. Imaging consisted of two overlapping bed positions to mitigate the triangular axial sensitivity profile of the scanner, each of 15 – 20 min duration, in 3D mode. Where ^90^Y was not available as a radionuclide selection in the acquisition software a long-lived isotope was selected (e.g. ^22^Na) to avoid any scanner decay correction and data were quantified after reconstruction by taking into account the ratio of the positron branching ratios of ^90^Y and the acquisition radionuclide. No additional sensitivity measures were required.

Sites were encouraged to use reconstruction parameters that had proven successful in their own ^90^Y experience, with all available corrections (scatter, attenuation, random coincidences, ToF and RR where available). Following day 7 of imaging a radiographic contrast agent was added to the background compartment of the phantom and a CT study performed to aid in image segmentation for volume definition of the fillable spheres.

### Image analysis

Data were transferred in DICOM format via a secure data server (ABX-CRO Advanced Pharmaceutical Services, Dresden, Germany) to the core laboratory in Sydney (Royal North Shore Hospital, Sydney, Australia) for consistent analysis. All analyses were performed by a single operator (K.W.) on a dedicated nuclear medicine workstation (HERMES; Nuclear Diagnostics, Stockholm, Sweden) using in-house software written in IDL (Exelis Visual Information Solutions, Boulder, CO). For the quantitative assessment the shipping certificate indicating the amount of ^90^Y in the initial vial as determined by the supplier was treated as the gold standard, and the fraction of residual measured in the vial during the experimental procedure was regarded as the total residual (possible residual in the syringe, needle, beaker etc. was considered negligible due to the difficulty in reliable measurement of ^90^Y in the dose calibrator). These measures, together with measured phantom volume, were used to derive the true concentration and activity in the phantom at each imaging time-point. The uncertainty in ‘true’ estimates of activity and concentration in the phantom was taken to be ±10 %, a combination of possible volume measurement error (<1 %) and uncertainty in the calibration of activity in the delivery vial.

**Fig. 1:**
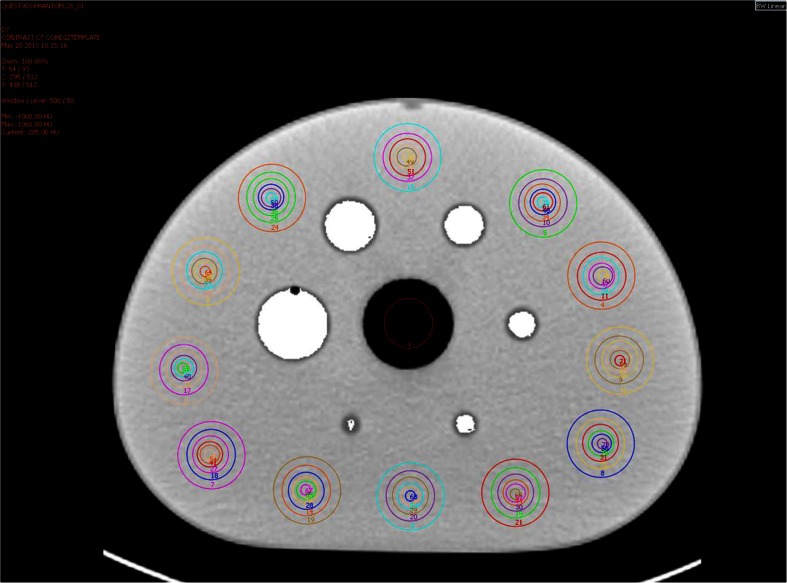
Transverse CT slice of a phantom showing segmented hot sphere VOIs (*white*), cold insert ROI, and 60 background ROIs corresponding to each sphere diameter as described in NEMA NU 2-2007
- Mean concentration for each of the CT-defined spherical VOIs (Fig. 1) and the associated recovery coefficient (RC) to assess partial volume effects (PVEs) on the day-0 data, defined as:
  1 $$ RC\left(\%\right)=\frac{\mathrm{Measured}\;\mathrm{Concentration}}{\mathrm{True}\;\mathrm{Concentration}}\times 100 $$
  Lines of best fit (*y* = 100 − *a*e^(−*bx*)^) for recovered concentration were compared with the curve obtained from repetition of an identical phantom procedure using ^18^F (Siemens mCT Biograph PET/CT, 3i21s 5-mm gaussian RR + ToF) processed in an identical manner to the experimental ^90^Y data to generate reference RCs.
  The change in recovery of the largest diameter hot sphere, least affected by PVEs, over the range of imaging days was assessed for consistency of recovery with deteriorating count statistics.
- Counts incorrectly misplaced in the central cold insert, assessed as the mean of counts in a central ROI replicated across five transverse slices (Fig. 1) as a percentage of true background concentration.
- Background variability (BV), in keeping with the recognized NEMA NU 2-2007 measure of image quality, was also explored as an indication of potential variation in background concentration measures as a result of poor image signal-to-noise ratio, defined as:
  2 $$ B{V}_s=\frac{STDE{V}_{B,s}}{C_{B,s}} $$
  where *C*_*B,s*_ is the average of the 60 background ROI counts for sphere size *s*, and *STDEV*_*B,s*_ is the standard deviation of the background ROI counts for sphere size *s*.

The coregistration of the reconstructed PET data from all imaging days with the contrast-enhanced CT study was confirmed and the CT data were used to segment the six fillable spheres as 3D volumes of interest (VOIs) using a semiautomated region-growing algorithm to delineate the physical sphere volume. Quantitative accuracy was assessed at each imaging time-point by measurement of:

- Total activity in the reconstructed field of view (FoV) as an indicator of total injected activity.
- Background concentration, following the NEMA NU 2-2007 guidelines (Fig. 1).

Given the number of contributing scanners and variations in submitted reconstruction parameters, analysis of the data was stratified by averaging the results according to the categories listed in Table 2.

**Table 2:** Reconstructions that contributed to the study categorized according to ToF and non-ToF systems from specific vendors

System	Reconstruction algorithm	Reconstruction parameters	Filter	Correction	Number of reconstructions
GE Healthcare ToF	3D OSEM	MLEM 24 – 72^a^	All-pass Gaussian	ToF, RR ToF ToF, RR ToF –	7 2 7 3 2
GE Healthcare non-ToF	2D OSEM 3D OSEM	MLEM 16 – 64^a^	All-pass Gaussian	– – RR – – –	4 5 2 3^f^ 5 4^f^
Philips ToF	BLOB OS TF^b^	4i8s 3i33s or 4i33s	–	ToF ToF	2 6
Philips non-ToF	3D RAMLA^c^		–	–	1
Siemens ToF	3D OSEM	1i21s 2i21s 3i21s	All-pass Gaussian All-pass Gaussian All-pass Gaussian	ToF, RR ToF, RR ToF, RR ToF, RR ToF, RR ToF, RR	9 3 4 2 5 5
Siemens non-ToF	2D OSEM 3D OSEM	MLEM 16 – 84^a^ ‘NETTRUES’^d^ ‘PROMPTS + RANDOMS’^e^		– RR RR	7 9 12

*ToF’* time-of-flight, *RR’* resolution recovery (point spread function recovery)

^a^Maximum likelihood expectation maximization “equivalent number” = no. of iterations × no. of subsets

^b^A list mode algorithm that uses spherical (‘blob’-shaped), as opposed to voxel-shaped, basis functions to enhance signal and suppress noise; the final image is produced through interpolation from overlapping blobs to voxels, which acts as a filter [35]

^c^Row-action maximum-likelihood algorithm that operates using a relaxation parameter to control the amount of correction applied in each iterative update [36]

^d^The acquisition mode on the Siemens Biograph series that performs direct subtraction of delayed coincidences event-by-event [37]

^e^The acquisition mode on the Siemens Biograph series that stores delayed coincidences as a separate acquisition for subtraction from prompt events at a later time [37]

^f^Denotes scanners with LYSO crystal material; all other scanners in this category (GE non-ToF) used BGO material

### Validation of methodology

At one site three consecutive scans were performed on the same phantom with the same scanner (Siemens mCT Biograph) using identical image acquisition and reconstruction parameters. Consistency in the above measures between the three scans was assessed to indicate uncertainty that might be expected due to random noise and variations. Furthermore, at a single site a lengthy 8-h acquisition (GE Healthcare Discovery 690, ^90^Y acquisition isotope) of the phantom was performed in addition to the standard 40-min acquisition, the data from which were used to look for any improvements with increased count statistics.

In addition, two datasets—one phantom study with ^18^F (to act as a reference, performed on a Siemens Biograph mCT, reconstructed using 3i21s ToF + RR and a 5-mm gaussian filter) and one phantom study with ^90^Y (performed on a GE Healthcare Discovery 690, quantified by the scanner, (i.e. performed with ^90^Y as the acquisition isotope, and reconstructed with 3i18s ToF and an all-pass filter)—were analysed by a physicist at an independent institution not involved in the study. Background concentration and hot sphere concentrations and recovery were measured using the following three software packages for comparison with the in-house QUEST method:

- An in-house ImageJ plug-in (NIH, Bethesda, MD) that uses NEMA guidelines to measure background concentration and the mean of a 50 % threshold-generated VOI to measure hot sphere recovery [25].
- Software provided as part of the European Association of Nuclear Medicine Research Ltd (EARL) initiative for standard image quality assessment of background concentration and hot sphere recovery as measured through threshold-derived VOIs on the central slice of the PET images [26].
- The ROVER package (ABX-CRO Advanced Pharmaceutical Services, Dresden, Germany) which again applies a 50 % growing algorithm to generate a VOI for hot sphere recovery measurement, and measures background concentration as the mean of two generated background VOIs, all of which are manually placed by the user.

## Results

A total of 47 centres from 13 countries contributed data to the study. The average total activity in the phantom at the first imaging time-point was 3.26 GBq, with a standard deviation of 0.26 GBq (8 %).

### Dose calibrator measures

The average absolute difference between an individual site’s measured ^90^Y activity in the delivery vial in the local dose calibrator and the vendor-supplied calibration certificate, decay-corrected to the same time-point, was 5 %, with a measured range of −4 – +25 %, and a median of +2.5 %.

### Quantitative assessment

**Fig. 2:**
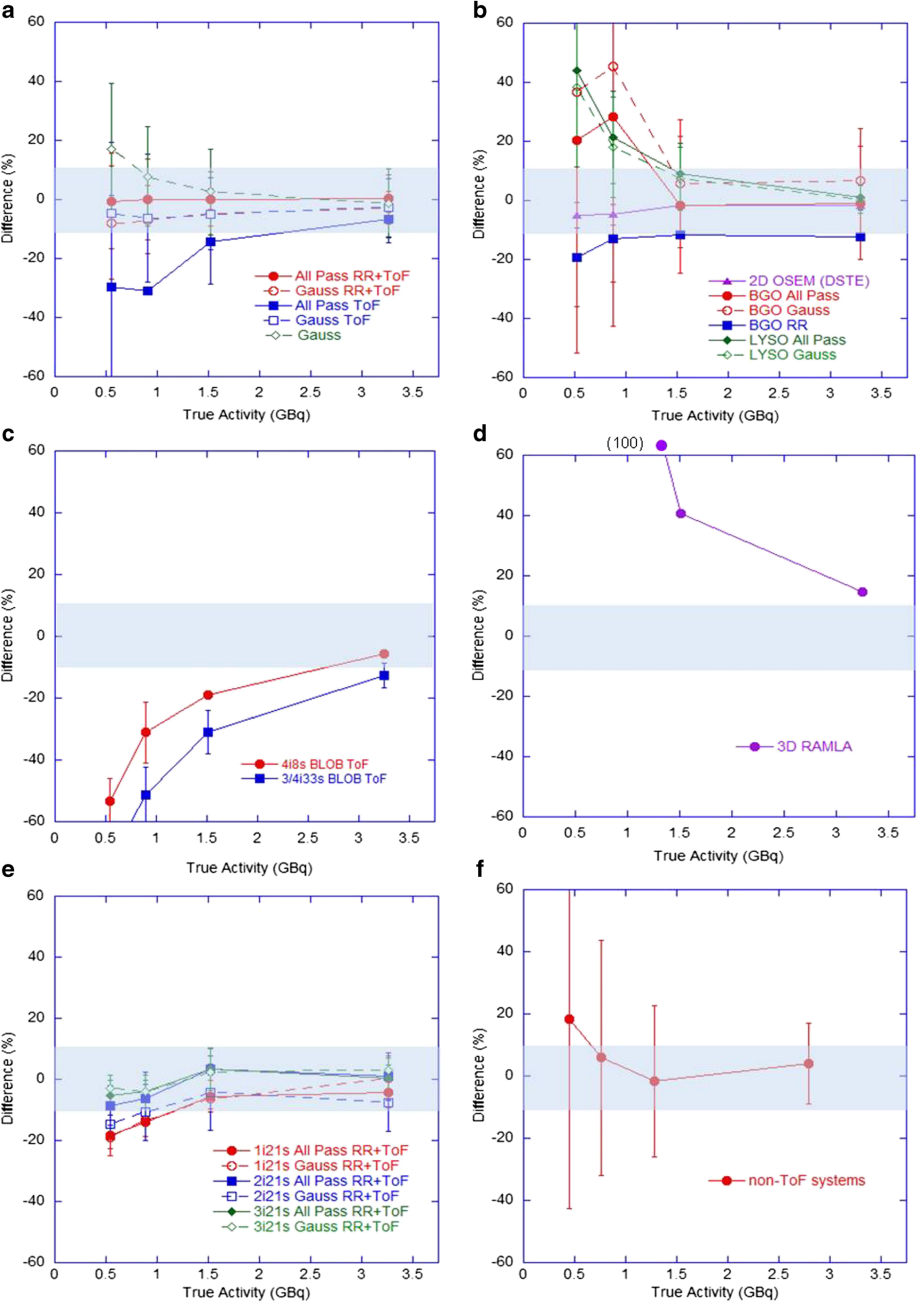
Differences in measured total activity in the FoV with respect to the expected total activity over all four imaging time points for (**a**) GE Healthcare ToF systems (*N* = 21), (**b**) GE Healthcare non-ToF systems (*N* = 23), (**c**) Philips ToF systems (*N* = 8), (**d**) Philips non-ToF systems (*N* = 1), (**e**) Siemens ToF systems (*N* = 28), (**f**) Siemens non-ToF reconstructions (*N* = 28, including only ‘PROMPTS + RANDOMS’ mode acquisitions,* see explanation in text*). Each datum is the mean for all scanners and the error bars represent one standard deviation (SD) of the measured values

**Fig. 3:**
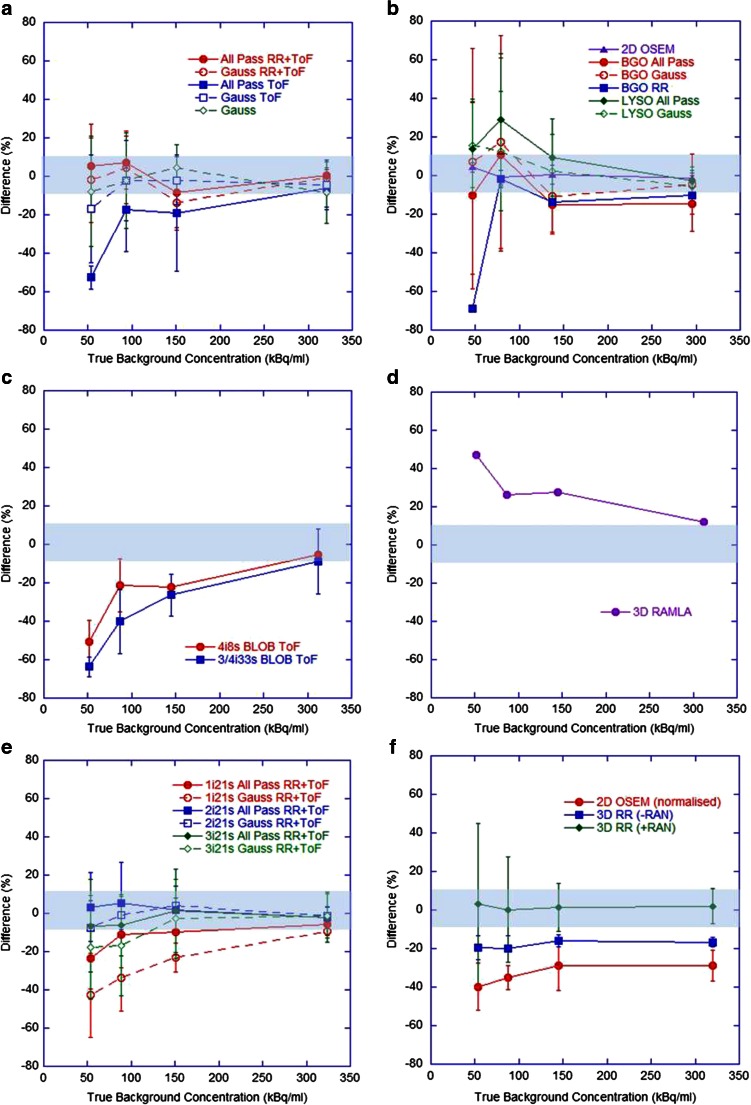
Differences in measured background concentration with respect to the true background concentration for (**a**) GE Healthcare ToF systems, (**b**) GE Healthcare non-ToF systems, (**c**) Philips ToF systems, (**d**) Philips non-ToF systems, (**e**) Siemens ToF systems, (**f**) Siemens non-ToF systems (where *+RAN* and *-RAN* correspond to data acquired in ‘PROMTS + RANDOMS’ and ‘NETTRUES’ mode, respectively, and where *-RAN* was normalized for analysis)

The accuracy of total activity measured in the FoV and the measured concentration of activity in the phantom background at each imaging time-point are shown in Figs. 2 and 3, respectively. Values are expressed as the percentage difference between the measured and expected values, where each measured value is the mean for a given category (note that the number of measured data that underlies these measured values does vary between scanner and reconstruction methods). The standard deviations of measures are shown as error bars (thus representing the combined inter-site variability and measurement error at consistent reconstruction parameters) and a general ±10 % tolerance is represented by the shaded region (representing expected uncertainty in ‘true’ values). For the Siemens non-ToF systems, only ‘PROMPTS + RANDOMS’ mode acquisitions were included (Fig. 2f) because acquisitions in ‘NETTRUES’ mode resulted in extremely large overestimates when quantification was performed through rescaling of the acquisition branching ratio, and the reconstructed data were therefore normalized to the actual total activity in the phantom, such that estimates of total activity were not meaningful. Despite this post hoc normalization approach not being ideal in a clinical scenario (due to the difficulty in measuring residual in the delivery apparatus and the potential for stasis to be reached during treatment), it was explored for comparison purposes in this controlled phantom study.

Current generation GE Healthcare and Siemens ToF systems with RR and an all-pass filter produced acceptable estimates (within ±10 %) of total activity and background concentration over the range 0.5 – 3 GBq and 50 – 300 kBq/ml, respectively, with improvements seen in Siemens systems when reconstructing with two or three iterations. No evidence of detector saturation was seen, in agreement with the literature [10, 12, 19, 20]. The non-ToF Siemens systems gave accurate estimates of background concentration when acquired in ‘PROMPTS + RANDOMS’ mode. Both BGO and LYSO non-ToF GE Healthcare systems showed similar behaviour, including overestimates of activity at levels below 1.5 GBq and estimates of background above 100 kBq/ml within 15 %. Philips Gemini ToF systems appeared to underestimate total activity at levels below 3 GBq and background concentrations below 300 kBq/ml, whilst large overestimates across the entire range were seen on the 3D RAMLA reconstruction. For high activity levels (about 3 GBq) in the FoV, all scanners were capable of producing satisfactory estimates, presumably due to the improved count statistics which allowed improved scatter modelling and reduced effect of randoms subtraction.

**Fig. 4:**
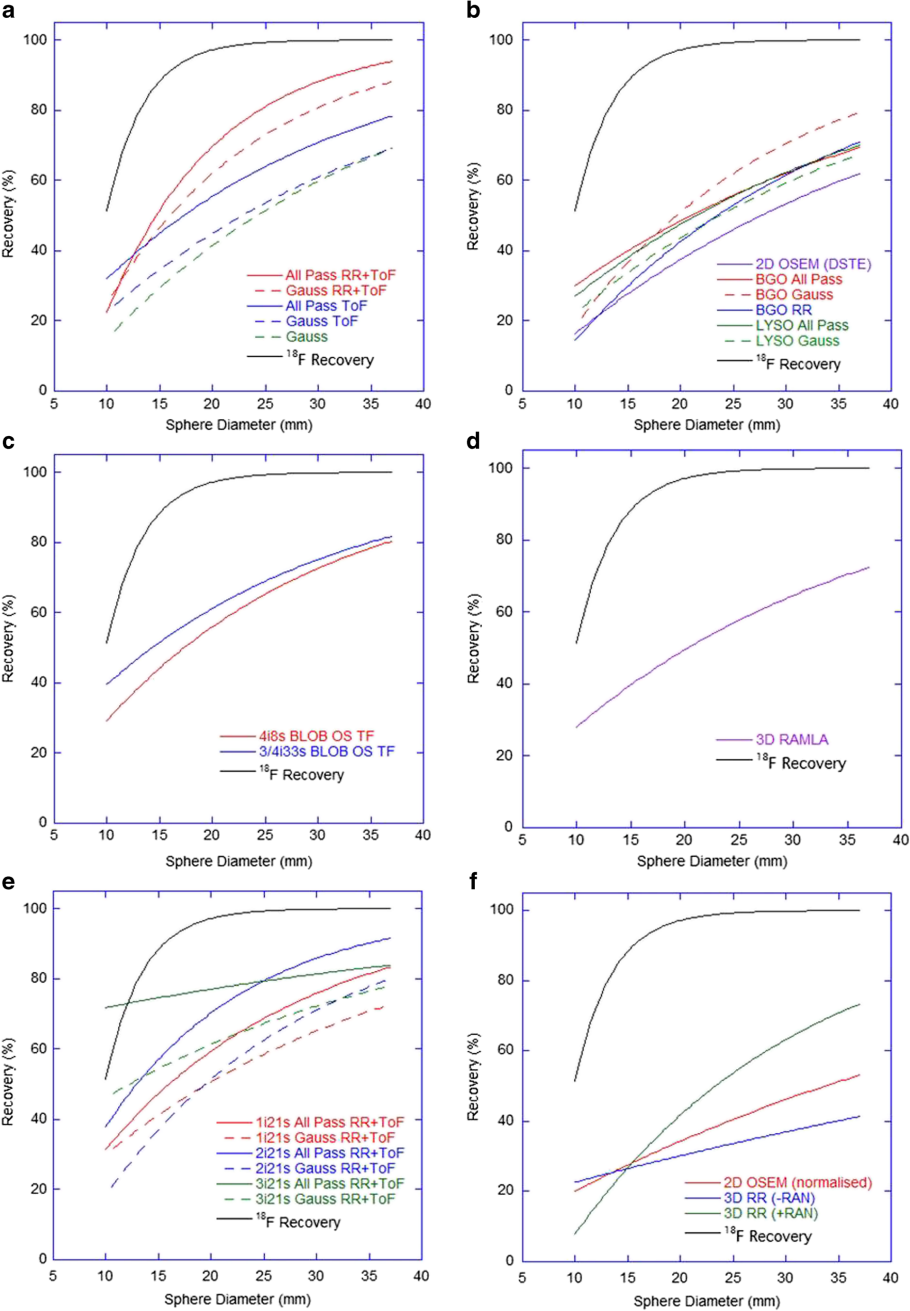
Lines of best fit (*y* = 100 − *a*e^(−*bx*)^) for recovered concentrations in hot spheres of various diameters on day-0 imaging for (**a**) GE Healthcare ToF systems (*R* ^2^ = 0.94 – 0.98), (**b**) GE Healthcare non-ToF systems (*R* ^2^ = 0.90 – 0.99), (**c**) Philips ToF systems (*R* ^2^ = 0.97 – 0.98), (**d**) Philips non-ToF systems (*R* ^2^ = 0.90 – 0.96), (**e**) Siemens ToF systems (*R* ^2^ = 0.80 – 0.99), (**f**) Siemens non-ToF systems (*R* ^2^ = 0.94 – 0.97) (where *+RAN* and *-RAN* correspond to data acquired in ‘PROMTS + RANDOMS’ and ‘NETTRUES’ mode, respectively, and where *-RAN* was normalized for analysis). The *black line* of reference is the recovery curve for ^18^F derived from the same experiment

**Fig. 5:**
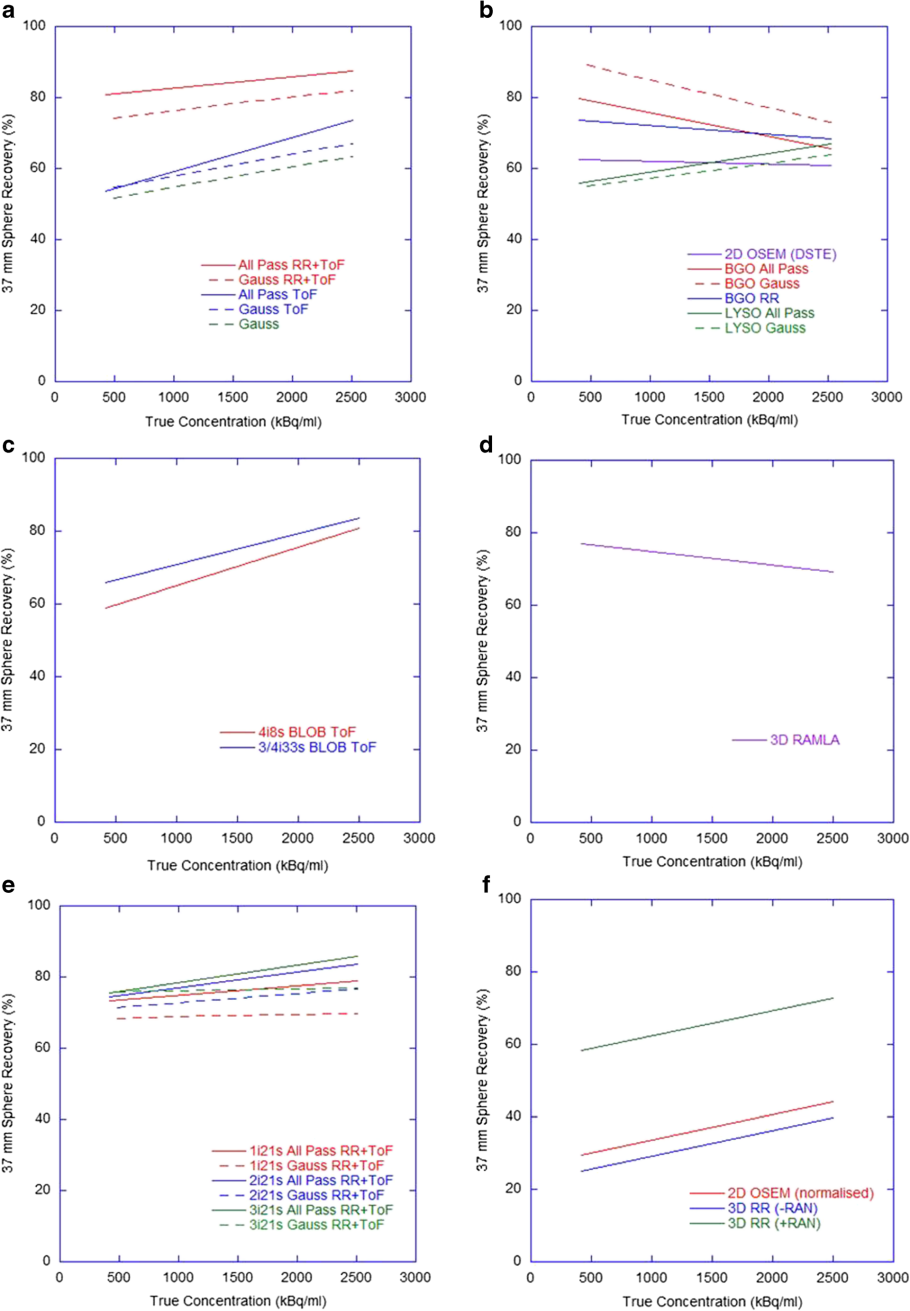
Lines of best fit (*y* = *a* + *bx*) for recovered concentrations in the largest hot sphere at different concentrations for (**a**) GE Healthcare ToF systems (*R* ^2^ = 0.54 – 0.87), (**b**) GE Healthcare non-ToF systems (*R* ^2^ = 0.31 – 0.93), (**c**) Philips ToF systems (*R* ^2^ = 0.55 – 0.88), (**d**) Philips non-ToF systems (*R* ^2^ = 0.47), (**e**) Siemens ToF systems (*R* ^2^ = 0.21 – 0.75), (**f**) Siemens non-ToF systems (*R* ^2^ = 0.77 – 0.99) (where *+RAN* and *-RAN* correspond to data acquired in ‘PROMTS + RANDOMS’ and ‘NETTRUES’ mode, respectively, and where *-RAN* was normalized for analysis)

Recovery of activity concentration measured in the hot spheres on day 0 of imaging is shown in Fig. 4, and the change in this recovery for the 37-mm diameter hot sphere over different days of imaging in Fig. 5. All ToF systems demonstrated comparable recovery of concentration in hot spheres (note that at this day-0 imaging time-point these reconstructions also had comparable measures of background concentration), and in all cases this was inferior to that achieved with ^18^F. Postreconstruction gaussian filtering resulted in a decrease in recovery due to smoothing of the activity concentration outside the geometrical volume. All systems demonstrated a steady decline in both ^90^Y and ^18^F recovery for spheres with a diameter below 37 mm due to PVE. The Siemens ToF reconstructions with two or three iterations were again superior to a single iteration. Whilst Siemens non-ToF data acquired in ‘PROMPTS + RANDOMS’ mode demonstrated improvement over ‘NETTRUES’ mode, with recovery similar to the GE Healthcare non-ToF reconstructions, the non-ToF systems generally achieved poorer recovery. All ^90^Y data suffered from underestimates in the range of 10 – 20 % of the true activity concentration of even the largest volume sphere (∅ 37 mm), a finding consistent with independent analyses of data (see Fig. 8). In ToF systems recovery underestimates for the largest hot sphere over all days of imaging gradually deteriorated (Fig. 5). This may have been due to the influence of the ^176^Lu present in the detector crystals, which has been suggested to affect low count studies [19]. Non-ToF systems demonstrated some variation, with a slightly better recovery with the BGO system and RAMLA at lower concentrations, which may have been due to deteriorating noise and associated spurious high counts in voxels.

**Fig. 6:**
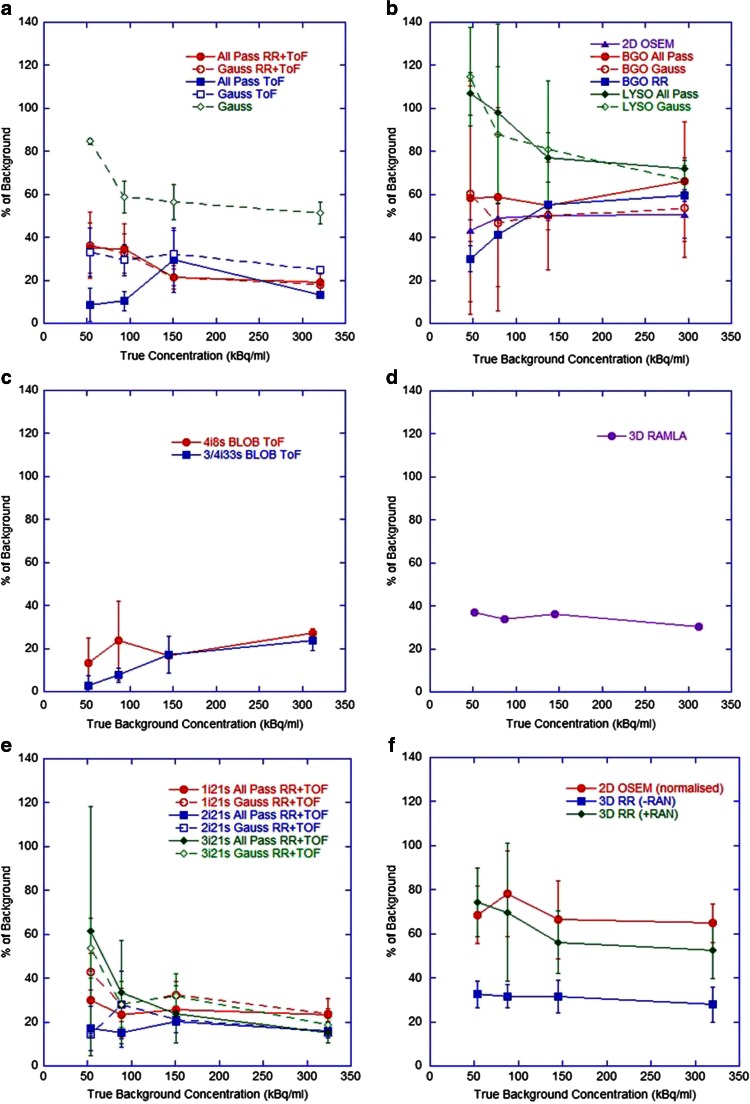
Measured activity concentrations in the cold insert as percentages of the true background concentrations at different concentrations for (**a**) GE Healthcare ToF systems, (**b**) GE Healthcare non-ToF systems, (**c**) Philips ToF systems, (**d**) Philips non-ToF systems, (**e**) Siemens ToF systems, (**f**) Siemens non-ToF systems (where *+RAN* and *-RAN* correspond to data acquired in ‘PROMTS + RANDOMS’ and ‘NETTRUES’ mode, respectively, and where *-RAN* was normalized for analysis). This measure predominantly reflects the accuracy of scatter and randoms corrections

The activity concentrations measured in the cold insert are displayed as percentages of true background concentrations on different days of imaging in Fig. 6. All ToF systems exhibited similar behaviour (on average about 30 % of background) and in general non-ToF systems measured far greater scattered events in the cold insert (on average about 60 % of background). For the GE Healthcare non-ToF systems this was reduced in BGO scanners, perhaps due to the absence of background counts from the natural ^176^Lu in the crystals.

**Fig. 7:**
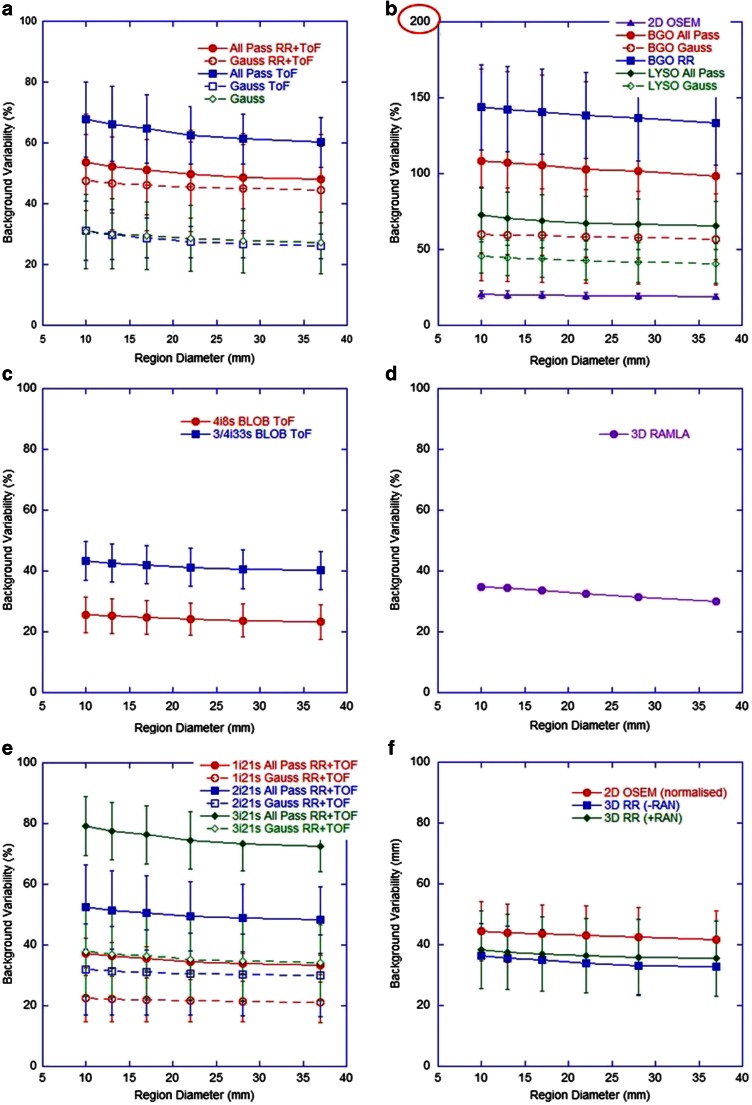
Background variability for different region diameters for (**a**) GE Healthcare ToF systems, (**b**) GE Healthcare non-ToF systems (note different scale on the *y*-axis), (**c**) Philips ToF systems, (**d**) Philips non-ToF systems, (**e**) Siemens ToF systems, (**f**) Siemens non-ToF systems (where *+RAN* and *-RAN* correspond to data acquired in ‘PROMTS + RANDOMS’ and ‘NETTRUES’ mode, respectively, and where *-RAN* was normalized for analysis)

Figure 7 shows the BV measured on day 0 using regions of various diameters corresponding to the diameters of each hot sphere. As expected, BV was improved with postreconstruction gaussian filtering and deteriorated with increasing numbers of iterations and associated noise. The ToF systems from all three vendors displayed similar behaviour, with slightly better data from the Philips system perhaps due to the noise suppression properties of the BLOB OS TF algorithm. The non-ToF GE Healthcare BGO systems displayed significantly poorer results than their LYSO counterparts. This may have been due to the larger coincidence timing window associated with BGO, which increases the random coincidence rate and may further increase noise in the reconstructed images.

As a measure of repeatability, the standard deviations between quantitative measures from three consecutive scans with an identical phantom, acquisition and reconstruction protocol are shown in Table 3. The total measured activity remained constant and a change in measured concentration in the order of 10 % was seen. The change in quantitative measures with the 8-h acquisition (single bed position) was within 10 % of that with the standard 40-min (two bed positions; Table 3), except for measures of misplaced events in the cold insert, where the improved counting statistics increased the cold contrast ratio by 20 %. This may have been due to better estimates of scatter prior to subtraction.

**Table 3:** Standard deviations between quantitative measures from identical processing of three consecutive scans performed on a Siemens mCT scanner (as percentages of true values), and the differences between quantitative measures from identical reconstructions of consecutive scans of 40-min duration and 8-h duration on a GE Healthcare Discovery 690 system (as percentages of those measured in the 8-h acquisition)

Measure	Standard deviation between three consecutive scans (%)	Difference between 40-min and 8-h acquisition (%)
Total activity	0.14	–^a^
Background concentration	13	−6
Cold region counts	10	+20
Recovery		
37-mm sphere	5	+3
28-mm sphere	4	+3
22-mm sphere	8	−3

^a^Entire phantom not in FoV

**Fig. 8:**
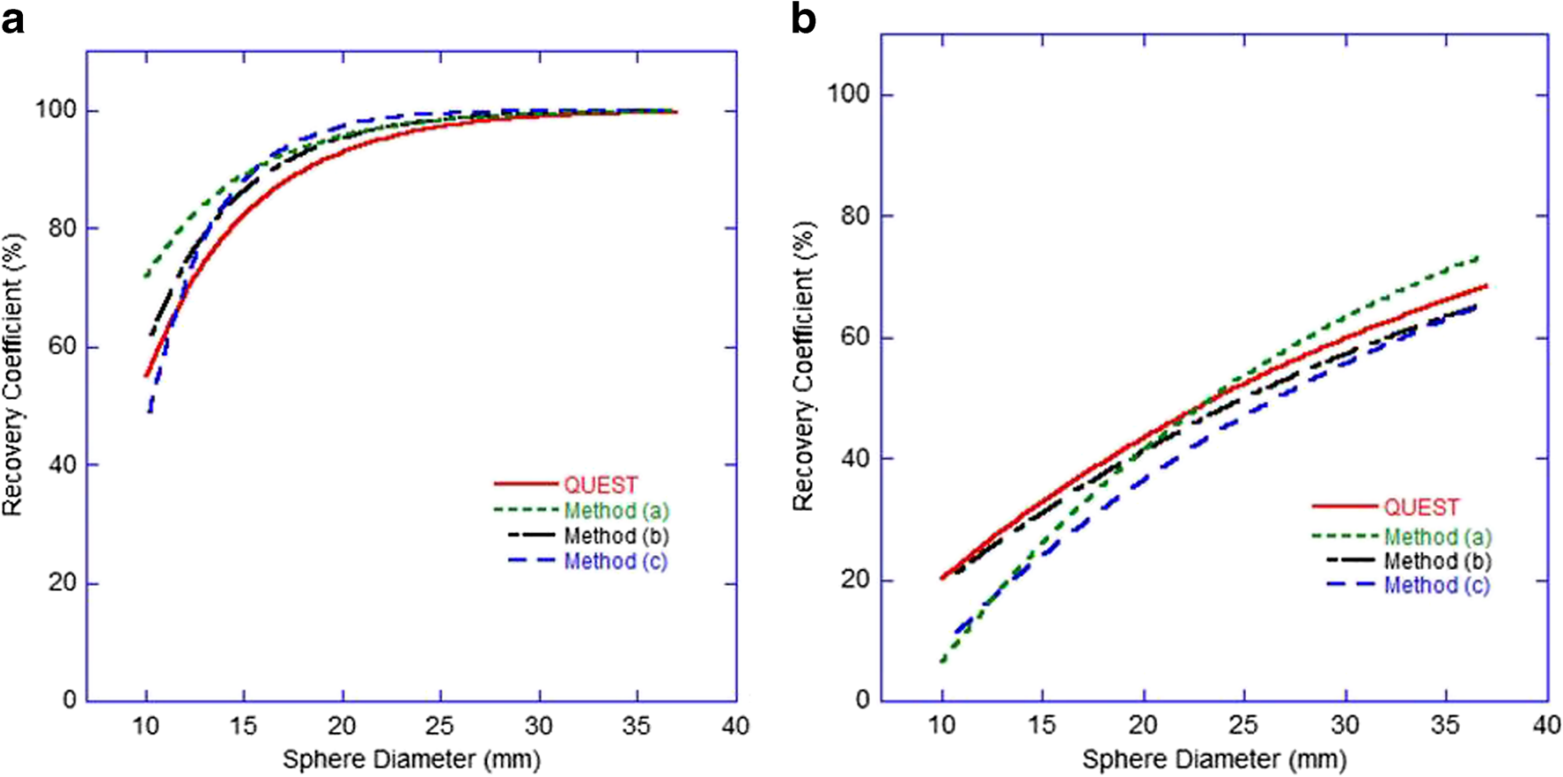
Recovered concentrations for hot spheres of various diameters using four software analysis methods for both ^18^F data (**a**) and ^90^Y data (**b**)

Comparison of the QUEST methodology with independent analysis using other software for a sample ^18^F and ^90^Y dataset are given in Table 4 for measures of background concentration and in Fig. 8 for measures of hot sphere recovery. All methods performed consistently when measuring both ^18^F and ^90^Y concentration data and demonstrated similar trends in underestimation in recovery curves. The ROVER package, method (c), produced slightly different measures of background concentration, most likely due to the use of a large VOI as opposed to multiple ROIs, and minor variations in recovery curves can be attributed to the method of VOI generation.

**Table 4:** Differences between measured and true values of background concentration for each of the analysis methods, represented as percentages of the true values

Method	Difference between measured and true background concentration (%)	
	^18^F	^90^Y
QUEST	9.9	5.1
Method (a)	9.9	3.9
Method (b)	9.8	1.9
Method (c)	6.6	11.3

## Discussion

It should be recognized that findings regarding scanner performance discussed in this work are not applicable to ^18^F imaging, only ^90^Y, and as such are not a reflection of scanner behaviour for the vast majority of clinical PET applications. Comparable and efficient scanner performance has been reported in the literature for qualitative and quantitative ^18^F imaging aspects for all vendors for example [27–29]).

The experimental protocol was chosen to cover a clinically realistic range of activities for resin microspheres, where a standard administration of ^90^Y SIR-Spheres for radioembolization is of the order of 1.6 GBq [30]. Given the diversity in ‘typical’ liver size and tumour burden, nonuniform deposition of microspheres, and the large differences in tumour targeting resulting from the superselective radioembolization procedure, pinpointing a representative concentration in background and hot spheres of the phantom to correspond to patient liver and tumour uptake is not straightforward. The literature expresses large differences in this respect, with tested sphere-to-background ratios ranging from 3:1 [19, 31] to 40:1 [12], and associated background concentrations from as low as 37 kBq/ml to as high as 470 kBq/ml [20]. Given the larger volume of the phantom compared to a human liver, a clinical scenario is thought to lie towards the higher end of the count spectrum explored in this study.

The achievable measures of total activity to within 10 % of expected values when using optimized reconstruction parameters on two out of three tested types of ToF systems implies the suitability of clinical ^90^Y PET for confirmation of delivered activity after radioembolization. This may be particularly useful when stasis is reached during administration, before the entire prescribed amount of microspheres has been implanted. This is also true of background activity concentration measures, which translates to absorbed dose estimates in nontarget liver. However, Fig. 6 implies that 20 – 40 % of this background level could be measured in adjacent true cold regions, which may lead to overestimation of absorbed dose in healthy liver regions that are devoid of any activity deposition. The difficulty in determining the existence of scatter and noise in reconstructed ^90^Y PET data versus true nontarget activity deposition in background regions was investigated by Kao et al. [5], with recommendation for qualitative assessment to rely on the pattern of uptake and conformation with underlying anatomy for extrahepatic queries, as opposed to relying on visual intensity.

Figures 2 and 3 highlight an apparent difference in behaviour between ToF systems from the different vendors. During discussions with the vendor Philips, it was suggested that the large underestimates at low count rates seen in the Gemini ToF reconstructions may have been due to the scatter correction algorithm used. Specifically, the magnitude of the scatter component may be underestimated at low count rates due to the fact that any negative pixels in the scatter subtracted sinogram are zeroed prior to subtraction (positivity constraint on the reconstruction algorithm), as demonstrated in the RAMLA reconstructed data (Fig. 3d). The current generation Philips ToF systems use the same approach to approximate the final scatter estimate which is then incorporated into list mode iterative reconstruction. As such, at the last iteration of ToF reconstruction, the scatter is estimated from the scatter under-corrected emission data (RAMLA results), resulting in an erroneously high scatter contribution, and hence leading to lower ToF emission counts at these low count rates. It should be noted that successful quantification of ^90^Y on Philips ToF PET scanners has been demonstrated in the literature, using a different approach to quantification that relies on a measured scanner-specific sensitivity factor [22]. This approach may be desirable on a single-site basis, but for the purpose of this work (as a precursor to a multicentre evaluation) it was not an ideal method due to the need for additional experimental work and the inability for all sites to have an identical approach to quantification.

There is an evident difference in both image quality and quantification when comparing scanners with and without ToF and RR. There is also a consistent underestimation in all quantitative measures in hot spheres, seen even at long acquisition times (Table 3), and under circumstances of accurate background quantification. This is most likely related to the excessive random and scatter events and low count rates when imaging ^90^Y, and the way in which the reconstruction algorithm deals with this, and is not evident for ^18^F data which benefits from a true count rate that is orders of magnitude greater than that of ^90^Y. Iterative reconstruction algorithms recover low frequency or background events first, implying that higher iterations are needed for accurate recovery of small hot objects. However, given the noise and low signal present in ^90^Y PET, higher iterations are not a practical solution, and this is demonstrated in Fig. 4 by the lack of an obvious improvement with an increasing number of iterations, and is in agreement with the literature [12, 20, 32].

 Furthermore, a longstanding problem in PET reconstruction is the bias introduced by the necessity to remove negative sinogram values (which become zeroed) following correction for random coincidences, in order to satisfy the assumption of a Poisson distribution which is the basis for expectation-maximization-based reconstructions, such as OSEM. This bias does not have a significant impact for the vast majority of clinical PET scanning. In the case of ^90^Y where extremely low count rates are observed in the setting of high random coincidences, the bias becomes greater. In contrast, it is known that as the fraction of random coincidences increases, the gain in signal to noise ratio associated with ToF increases [33], implying that current generation scanners that employ ToF would be more suited to imaging ^90^Y than previous generation scanners, an assumption that seems to be verified by these results. In addition, it is known that iterative reconstruction converges faster with the use of ToF [27]. These findings are also supported by other publications [12, 19, 34].

The importance of the treatment of random coincidences is well demonstrated in the Siemens data. The significant differences in quantification between non-ToF acquisitions in ‘PROMPTS + RANDOMS’ mode versus ‘NETTRUES’ mode is due to the fact that the latter performs direct subtraction of delayed coincidences event-by-event, as opposed to storing the separate acquisition of delayed coincidences that allows smoothing prior to subtraction from the prompt events. Direct subtraction without smoothing is more likely to result in false-negative values in the sinogram, which when reconstructed using the positivity constraint applied in OSEM algorithms creates noisy data and inaccurate quantification. The GE Healthcare systems employ the single-event based method of randoms correction (calculating the mean random coincidence rate for each line of response based on the coincidence timing window and the single photon event rate) and the non-ToF GE Healthcare systems do not exhibit the same extreme underestimates as seen in the non-ToF Siemens systems prior to the use of smoothing. Furthermore, scatter correction in low count studies may well be less accurate due to the difficulty of estimating scatter from such noisy sinograms, evident in the Philips data, and as suggested by van Elmbt et al. [19], may be further affected by additional signal coming from pair production in the LSO/LYSO crystals. The cumulative effect is a remarkable improvement in the ability of current generation scanners to image and quantify ^90^Y.

Results suggest that previous generation scanners without RR and ToF do not produce consistent quantitative ^90^Y measures for comparison with current generation scanners. From the range of data investigated in this study, ^90^Y imaging performance appears to be optimal for Siemens systems using two iterations and 21 subsets with ToF and RR for best quantification without compromising measures affected by noise, with an all-pass filter (or with a 5 – 8 mm gaussian filter for qualitative purposes). For GE Healthcare systems the use of an all-pass filter in conjunction with RR and ToF gave the most consistent results for quantification, and a subset analysis of data (not shown) suggested two iterations and 24 subsets. Investigation of the Philips ToF reconstructions of ^90^Y is ongoing, including communication with the vendor, but at present total measures of activity and concentration in background regions may be underestimated for low concentration regions.

In the imaging of ^90^Y for quantitative purposes with non-ToF generation GE Healthcare and Siemens scanners, measures of large areas of concentration (about 300 kBq/ml) can be expected, on average, to be within 9 % and 2 % of true values, respectively, but recovery of concentration measures in hot lesions (about 2,500 kBq/ml) can be expected to be inferior to imaging with their ToF counterparts, with average underestimates of −34 % and −27 %, respectively, for a 37-mm diameter object (see Table 5 for complete comparison). A different analysis method, such as a threshold-based VOI, may improve these RCs, but given the very noisy nature of the ^90^Y reconstructions this may also be affected by a spurious maximum value. This study suggests that with Siemens non-ToF scanners, data should not be acquired in ‘NETTRUES’ mode if correct quantification of ^90^Y is desired, and the most consistently accurate results were seen when using ‘PROMPTS-RANDOMS’ mode with two iterations and 21 subsets in combination with RR.

**Table 5:** Reconstruction parameters that provided most accurate quantification over the assessments performed in this investigation, and the expected accuracy and standard deviations associated with measures of warm background and hot spheres for each. All measures are based on the results from day-0 imaging, where phantom background and hot sphere concentration were about 300 kBq/ml and 2,500 kBq/ml, respectively

Vendor	Model	Recommended reconstruction for quantitative purposes	Error in warm background concentration measures (%)		Error in 37-mm hot sphere concentration measures (%)	
			Average ± SD	Range	Average ± SD	Range
GE Healthcare	Discovery 600 Discovery ST (E) Discovery RX	3D OSEM with all-pass filter: e.g. 2i24s	−9 ± 10	+4 – 29	−34 ± 9	−14 – 49
GE Healthcare	Discovery 690 Discovery 710	3D OSEM with all-pass filter: e.g. 2i24s + RR + ToF	1 ± 4	+6 – 7	−14 ± 9	−5 – 28
Philips	Gemini TF	3D OSEM (BLOB OS TF) with no filter: 4i8s + ToF	−5 ± 2	−4 – 6	−22 ± 3	−20 – 24
Siemens	Biograph (various)	3D OSEM with all-pass filter: e.g. 2i21s + RR (acquired in ‘PROMPTS + RANDOMS’ mode)	2 ± 9	+9 – 22	−27 ± 5	−20 – 40
Siemens	Biograph mCT	3D OSEM with all-pass filter: 2i21s + RR + ToF	−2 ± 6	+4 – 9	−16 ± 4	−13 – 22

A ±10 % uncertainty can be expected on quantitative measures due to random noise in the acquisition and reconstruction process, which is approximately consistent in regions of non-zero background activity and hot spots (Table 3). Coupled with the uncertainty in the ^90^Y activity (±10 %) treated as the gold standard in this work, quantitative measures on ToF PET systems with the reconstructions discussed can be expected to produce acceptable estimates of activity and concentration in large homogeneous areas over a clinically realistic range of values. It should be expected that hot lesion quantification (and so absorbed dose estimates) may be underestimated with all current generation scanners to a consistent degree of 15 – 20 % for a 37-mm diameter object. Such underestimates may be improved with a different volume definition technique, as explored by Goedicke et al. [22]. Given the lack of significant improvement in warm and hot volume quantification with increased acquisition durations (Table 3), a 40-min acquisition is recommended in the clinical setting, acquired as two bed positions (20 min each) to avoid the area of interest (liver) imposing on the edges of the FoV where noise is greatest in the reconstructed data, and to avoid peaking of the scanner’s sensitivity profile.

### Conclusion

In summary, current generation ToF PET scanners are capable of producing comparable quantification of ^90^Y over a large range of clinically realistic activity and concentration levels. In terms of quantitative accuracy of estimates and expected uncertainties for translation to clinical measures of absorbed dose, Table 5 shows the average errors and ranges of measures for those reconstructions that were found to be best, based on the data investigated in this work. Considering possible acceptance criteria for scanners acquiring data in a clinical trial setting, an achievable accuracy of concentration measures in large uniform regions of activity of 10 % (average) over a range of clinically realistic true concentrations (50 – 300 kBq/ml) may be considered suitable performance.

## Appendix: QUEST Investigators’ Team

Kathy P. Willowson · Michael J. Tapner · Hojjat Ahmadzadehfar · Holger Amthauer · Javier Arbizu · Ali A. Attarwala · Oreste Bagni · Francois Benard · Faustino Bonutti · Francesca Botta · Jan A. Boucek · Austin C. Bourgeois · Yong C. Bradley · Hans-Georg Buchholz · Karen-Anett Büsing · Thomas Carlier · Anna Celler · Morgan Cervo · Simona Civollani · Maurizio Conti · Allison J. Craig · Marta Cremonesi · Marco D’Andrea · Marco D’Arienzo · Yves D’Asseler · Fabio Di Martino · Mohan Doss · Heying Duan · Thomas Eugene · Mahila Ferrari · Luca Filippi · Patrick Flamen · Alison M. Fletcher · Glenn D. Flux · Eugene Fourkal · Ros Francis · Leonard M. Freeman · Onelio Geatti · Gerhard Glatting · Andreas Goedicke · Oliver S. Groβer · Cicero M.R. Habito · Aida Hallam · Sarah Heard · Martha Hoffmann · Søren Holm · Claire A. Hooker · Giuseppe Iaccarino · Stephen P. Jeans · Patricia Judy · Peter J. Julyan · Levent Kabasakal · Hans-Jürgen Kaiser · S. Cheenu Kappadath · Bieke Lambert · Michael Lassmann · Martin W. Law · Victor H. Lee · Francesca Leek · Renaud Lhommel · Martin A. Lodge · Alfredo Lopez · Markus Luster · Josep M. Martí-Climent · Daniel R. McGowan · J. Mark McKinney · Brian McLamb · Matthias Miederer · Flavia Molina-Duran · Stephen C. Moore · Darren G. Morgen · Jann Mortensen · Felix M. Mottaghy · Robert U. Mulder · Ole L. Munk · Razi Muzaffar · Sherry C. Ng · Kuldip S. Nijran · Robin de Nijs · Graeme J. O’Keefe · Medhat M. Osman · Jinsong Ouyang · Alexander S. Pasciak · Cinzia Pettinato · Robert A. Pooley · Ivo F. Rausch · Marion Reindl · Macarena Rodriguez-Fraile · Susanne Schlögl · Stefan O. Schönberg · Arif Sheikh · Lidia Strigari · Wendy Siman · Na Song · Shyam M. Srinivas · Peter F. Staanum · James R. Stone · Handan Tanyildizi · David J. Towey · Bruno Vanderlinden · Graeme Weir · Naichang Yu · Dale L. Bailey.

K.P. Willowson

Institute of Medical Physics, School of Physics, University of Sydney, NSW, Australia

kathy.willowson@sydney.edu.au

M.J. Tapner

Research and Development, Sirtex, Sydney, Australia

H. Ahmadzadehfar

Department of Nuclear Medicine, University Hospital Bonn, Bonn, Germany

H. Amthauer · O.S. Groβer

Department of Radiology and Nuclear Medicine, University Hospital Magdeburg, Magdeburg, Germany

J. Arbizu · J. M. Martí-Climent · M. Rodriguez-Fraile

Department of Nuclear Medicine, Clinica Universidad de Navarra, Pamplona, Spain

A.A. Attarwala · G. Glatting · F. Molina-Duran

Medical Radiation Physics/Radiation Protection, Universitätsmedizin Mannheim, Medical Faculty Mannheim, Heidelberg University, Mannheim, Germany

O. Bagni · L. Filippi

PET-CT Unit, Department of Nuclear Medicine, Santa Maria Goretti Hospital, Latina, Italy

F. Benard · A. Celler

Department of Radiology, Faculty of Medicine, University of British Columbia, Vancouver, Canada

F. Bonutti

Medical Physics Department, Academic Hospital of Udine, Italy

F. Botta · M. Ferrari

Medical Physics Unit, Istituto Europeo di Oncologia, Milano, Italy

J.A. Boucek · R. Francis

Department of Nuclear Medicine/PET, Sir Charles Gairdner Hospital, Nedlands, Australia

A.C. Bourgeois · Y.C. Bradley · A..S. Pasciak

University of Tennessee Graduate School of Medicine, Knoxville, TN, USA

H-G. Buchholz · M. Miederer

Department of Nuclear Medicine, University Medical Centre Mainz, Germany

K-A. Büsing · S. O. Schönberg

Institute of Clinical Radiology and Nuclear Medicine, University Medical Center Mannheim, Medical Faculty Mannheim, Heidelberg University, Mannheim, Germany

T. Carlier · T. Eugene

Nuclear Medicine Department, University Hospital of Nantes, Nantes, France and CRCNA—UMR 892 INSERM, 6299 CNRS, University of Nantes, Nantes, France

M. Cervo · S.C. Moore

Department of Radiology, Brigham & Women’s Hospital and Harvard Medical School, Boston, MA, USA

S. Civollani · C. Pettinato

Medical Physics Unit, AOU S.Orsola Malpighi, Bologna, Italy

M. Conti

Siemens Healthcare Molecular Imaging, Knoxville, TN, USA

A.J. Craig · G.D. Flux

Joint Department of Physics, Royal Marsden Hospital & Institute of Cancer Research, Sutton, UK

M. Cremonesi

Radiation Research Unit, Istituto Europeo di Oncologia, Milano, Italy

M. D’Andrea · G. Iaccarino · L. Strigari

Laboratory of Medical Physics and Expert System, Regina Elena National Cancer Institute, Rome, Italy

M. D’Arienzo

ENEA, National Institute of Ionizing Radiation Metrology, Roma, Italy

Y. D’Asseler · B. Lambert

Nuclear Medicine, Ghent University Hospital, Gent, Belgium

F. Di Martino

U.O.Fisica Sanitaria, Azienda Ospedaliero-Universitaria Pisana, Pisa, Italy

M. Doss

Diagnostic Imaging, Fox Chase Cancer Center, Philadelphia, PA, USA

H. Duan · M. Hoffmann

Department of Biomedical Imaging and Image-guided Therapy, Division of Nuclear Medicine, Medical University of Vienna, Austria

P. Flamen · B. Vanderlinden

Department of Nuclear Medicine, Institut Bordet, Université Libre de Bruxelles, Brussels, Belgium

A.M. Fletcher

Clinical Research Imaging Centre, University of Edinburgh, Edinburgh, UK

E. Fourkal

Department of Radiation Oncology, Fox Chase Cancer Center, Philadelphia, PA, USA

L.M. Freeman

Division of Nuclear Medicine, Montefiore Medical Center (Moses), New York, NY, USA

O. Geatti

Nuclear Medicine Department, Academic Hospital of Udine, Italy

A. Goedicke

Department of Nuclear Medicine, University Hospital, Aachen, Germany

Oncology Solutions Department, Philips Research Laboratories, High Tech Campus, Eindhoven, The Netherlands

C.M.R. Habito · J. Ouyang

Department of Radiology, Massachusetts General Hospital, Harvard Medical School, Boston, MA, USA

A. Hallam · Darren G. Morgan

Oxford University Hospitals, Oxford, UK

S. Heard · F. Leek

Addenbrooke’s Hospital, Cambridge University Hospitals NHS Foundation Trust, Cambridge, UK

S. Holm · J. Mortensen · R. de Nijs

Department of Clinical Physiology, Nuclear Medicine and PET, Rigshospitalet, Copenhagen University Hospital, Copenhagen, Denmark

C.A. Hooker

Department of Nuclear Medicine, King’s College Hospital NHS Foundation Trust, London, UK

S.P. Jeans · P.J. Julyan

Christie Medical Physics and Engineering, The Christie NHS Foundation Trust, Manchester, UK

L Kabasakal · H. Tanyildizi

Cerrahpasa Medical Faculty, Istanbul University, Istanbul, Turkey

H. Tanyildizi

Department of Nuclear Physics, Science Faculty, Istanbul University, Istanbul, Turkey

S.C. Kappadath · W. Siman

Department of Imaging Physics, The University of Texas MD Anderson Cancer Center, Houston, Texas, USA, & The University of Texas Graduate School of Biomedical Sciences, Houston, Texas, USA

M. Lassmann · S. Schlögl

Department of Nuclear Medicine, University Würzburg, Würzburg, Germany

M.W. Law

Department of Radiology, Queen Mary Hospital, Hong Kong

V.H. Lee · S.C. Ng

Department of Clinical Oncology, Li Ka Shing Faculty of Medicine, The University of Hong Kong. Hong Kong

R. Lhommel

Nuclear Medicine, Molecular Imaging, Radiotherapy, and Oncology Unit (MIRO), IECR, Université Catholique de Louvain, Brussels, Belgium

M.A. Lodge

Division of Nuclear Medicine, Russell H Morgan Department of Radiology and Radiological Sciences, Johns Hopkins University School of Medicine, Baltimore, Maryland, USA

M. Luster

Department of Nuclear Medicine, University Hospital Marburg, Marburg, Germany

D.R. McGowan

Radiation Physics and Protection, Oxford University Hospitals NHS Trust, Oxford, UK & Department of Oncology, University of Oxford, Oxford, UK

B. McLamb

School of Nuclear Medicine Technology, University of North Carolina Hospitals, Chapel Hill, NC, USA

H-J. Kaiser · F.M. Mottaghy

Department of Nuclear Medicine, University Hospital RWTH Aachen University, Aachen, Germany

R.U. Mulder · Patricia Judy · James R. Stone · Alfredo Lopez

Radiology Medical Physics & Interventional Radiology & Department of Nuclear Medicine, University of Virginia Health System, Charlottesville, VA, USA

O.L. Munk · P.F. Staanum

Department of Nuclear Medicine & PET Centre, Aarhus University Hospital, Denmark

R. Muzaffar · M.M. Osman

Nuclear Medicine and PET/CT, Saint Louis University, Saint Louis, MO, USA

K.S. Nijran · D.J. Towey

Radiological Sciences Unit, Imperial College Healthcare NHS Trust, London, UK

G.J. O’Keefe

Department of Nuclear Medicine, Austin Health, Melbourne, Australia

R.A. Pooley · J.M. McKinney

Department of Radiology, Mayo Clinic College of Medicine Jacksonville, FL, USA

I.F. Rausch

Centre for Medical Physics and Biomedical Engineering, Medical University of Vienna, Vienna, Austria

M. Reindl

Institute of Diagnostic and Interventional Radiology, Neuroradiology and Nuclear Medicine, Klinikum Bogenhausen, Munich, Germany

A. Sheikh

University of North Carolina, Chapel Hill, NC, USA

N. Song

Division of Nuclear Medicine, Department of Radiology, Montefiore Medical Center, New York, NY, USA

S.M. Srinivas

Department of Nuclear Medicine, Imaging Institute, & Center for PET and Molecular Imaging, Staff Nuclear Medicine, Adjunct Faculty in Dept. of Biomedical Engineering at CSU, Cleveland Clinic, Cleveland, OH, USA

G. Weir

Royal Infirmary of Edinburgh, Edinburgh, UK

N. Yu

Department of Radiation Oncology, Cleveland, OH, USA

D.L. Bailey

Department of Nuclear Medicine, Royal North Shore Hospital, Sydney, Australia

Medical Radiation Science, Faculty of Health Science University of Sydney, Sydney, Australia
